# KLF14 and SREBF-1 Binding Site Associations with Orphan Receptor Promoters in Metabolic Syndrome

**DOI:** 10.3390/ijms26072849

**Published:** 2025-03-21

**Authors:** Julio Jesús Garcia-Coste, Santiago Villafaña-Rauda, Karla Aidee Aguayo-Cerón, Cruz Vargas-De-León, Rodrigo Romero-Nava

**Affiliations:** 1 Laboratorio de Investigación en Genética de Enfermedades Metabólicas, Escuela Superior de Medicina, Instituto Politécnico Nacional, Ciudad de Mexico 11340, Mexico; jjcoste18@gmail.com (J.J.G.-C.); aidee.aguayo@gmail.com (K.A.A.-C.); 2 Laboratorio de Modelación Bioestadística para la Salud, Escuela Superior de Medicina, Instituto Politécnico Nacional, Ciudad de Mexico 11340, Mexico; 3 Laboratorio de Terapia Génica Experimental, Escuela Superior de Medicina, Instituto Politécnico Nacional, Ciudad de Mexico 11340, Mexico; santiagovr1@gmail.com; 4 División de Investigación, Hospital Juárez de México, Ciudad de México 07760, Mexico

**Keywords:** metabolic syndrome, transcription factors, KLF14, SREBF-1, orphan receptors, gene regulation, bioinformatics, sequence analysis

## Abstract

This study investigated the relationship between the transcription factors (TFs) KLF14 and SREBF-1 and orphan receptors (ORs) in the context of metabolic syndrome (MetS). A detailed bioinformatics analysis identified a significant association between the presence of binding sites (BS) for these TFs in the promoters of ORs genes and the total number of BS in the distal region. The results suggest that KLF14 and SREBF-1 can regulate the expression of some of these genes and, in turn, can modulate the development of MetS. Although a stronger association was observed with KLF14, both factors showed a significant contribution. Additionally, the sequence similarity of KLF14 also contributed to the quantity of BS in the gene’s distal region (DR). The statistical models used, such as Poisson and negative binomial regression, confirmed these associations and allowed for the appropriate adjustment of overdispersion present in the data. However, no significant differences in receptor groups (orphan G Protein-Coupled Rereptors (oGPCRs) and G Protein-Coupled Receptors associated with MetS (GPCRs-MetS)) regarding their relationship with TFs were found. In conclusion, this study provides strong evidence of the importance of KLF14 and SREBF-1 in regulating orphan receptors genes and their participation in the development of metabolic syndrome.

## 1. Introduction

Successful progress in eradicating many infectious diseases has led to a significant increase in non-communicable diseases (NCDs), making them the leading cause of morbidity and mortality globally. Among these diseases, metabolic syndrome (MetS) has emerged as a critical issue. It is important to note that metabolic syndrome is not a single disease but rather a combination of risk factors that predispose individuals to cardiovascular diseases and other metabolic disorders [1]. These elements make it a global public health problem [2], and it is equally defined as a metabolic disorder with significant incidence, directly related to chronic non-communicable diseases that cause high morbidity and mortality and includes central obesity, elevated triglycerides, atherogenic dyslipidemia, hyperglycemia, and hypertension [3]. Several international organizations have attempted to diagnose metabolic syndrome according to different consensus and diagnostic criteria, among which the National Cholesterol Education Program—Adult Treatment Panel III (NCEP-ATP III), the World Health Organization (WHO), the International Diabetes Federation (IDF), and the European Group for the Study of Insulin Resistance (EGIR) stand out [4].

In 2009, experts from the Israel Defense Forces (IDF) and the American Heart Association—National Heart, Lung, and Blood Institute (AHA/NHLBI) Adult Treatment Panel III issued guidelines that unified the diagnostic criteria for metabolic syndrome after researching potential etiological factors. According to the Harmonizing the Metabolic Syndrome criteria, the diagnosis of metabolic syndrome is established by several indicators. These include increased waist circumference, which has a specific definition based on the population and country; elevated triglycerides, defined as levels greater than or equal to 150 mg/dL (or on specific lipid-lowering treatment); decreased High-Density Lipoprotein (HDL) cholesterol, which is less than 40 mg/dL in men or less than 50 mg/dL in women (or undergoing treatment affecting HDL levels); elevated blood pressure, with systolic blood pressure greater than or equal to 130 mmHg and/or diastolic blood pressure greater than or equal to 85 mmHg (or on antihypertensive treatment); and elevated fasting glucose, defined as levels greater than or equal to 100 mg/dL (or on medication for elevated glucose) [5]. According to the above, MetS is identified with at least three of the five proposed components.

The incidence of MetS is linked to obesity and type 2 diabetes. According to the National Health and Nutrition Examination Survey (NHANES) data from 1988 to 2010, in the United States (US), body mass index (BMI) increased annually by 0.37% in both men and women, and waist circumference also increased. In 2017, the Center for Disease Control and Prevention (CDC) reported that approximately 30.2 million adults in the US had type 2 diabetes, with one-quarter unaware of it. The incidence of diabetes increases with age, primarily affecting older adults, and the prevalence of prediabetes or metabolic syndrome is significantly higher, affecting about one-third of adults in the US [1]. MetS prevalence in Mexican adults, using the harmonized definition, increased substantially from 2006 to 2018. The prevalence rose from 40.2% in 2006 to 56.31% in 2018, representing a 20.22% increase overall. Women consistently had higher MetS prevalence rates than men. Abdominal obesity was the most common MetS component, with prevalence exceeding 70% throughout the study period. Hypertriglyceridemia showed the largest percentage increase, rising by 61.85% over the study period [6,7,8]. In the Mexico City Diabetes Study, 39.9% and 59.9% prevalence rates were reported for men and women, using the NCEP-ATPIII criteria [9]. In a study spanning more than 10 years, no significant increase in the prevalence of metabolic syndrome was observed despite the increase in abdominal obesity. In a subsequent report by the same group in Mexico City, prevalence rates of 31.9% and 54.4% were found using different criteria, attributed to a stricter definition of abdominal obesity in the latter case.

The transcription factors KLF14 and SREBF-1 are key in metabolic syndrome due to regulating lipid and glucose metabolism genes. KLF14 activates insulin and glucose genes in the liver, influencing gluconeogenesis. KLF14 expression is affected by diet, implying its role in altered metabolic processes in metabolic syndrome [10]. This transcription factor is a member of the Krüppel-like family of transcription factors, responsible for modulating gene expression in mammals [11,12]. KLFs contain conserved C2H2-type zinc finger domains at their C-termini, which bind to GC-rich sites in the regulatory regions of target genes. In the absence of experiments showing the direct binding of KLFs to the enhancers and promoters of target genes, common transcriptional targets have been predicted using the structural similarities between KLFs [13]. On the other hand, lipotoxicity can trigger type 2 diabetes, obesity, and insulin resistance [14]. SREBF-1 is a key transcription factor in the regulation of lipid synthesis and lipid homeostasis. It regulates the expression of genes involved in the synthesis of cholesterol, fatty acids, and triglycerides. Similarly, it acts as an activator transcription factor that binds to specific DNA sequences to regulate gene expression. In MetS, SREBF-1 may be involved in the metabolic dysfunction associated with lipid accumulation in tissues and insulin resistance [15]. Studies evaluating the expression of SREBF-1 in response to dietary and genetic manipulation in animals have provided additional strong evidence that they are involved in both lipogenesis and cholesterol homeostasis [16]. Specific analyses of individual isoforms suggest that SREBF-1 may selectively participate in activating genes involved in fatty acid metabolism and de novo lipogenesis [17].

G protein-coupled receptors (GPCRs) are crucial in cell signaling and participate in a wide range of physiological and pathological processes [18]. These receptors trigger cellular responses by binding to various ligands, such as hormones, neurotransmitters, and growth factors. In the context of metabolic syndrome, it has been suggested that certain GPCRs may be involved in the pathophysiology of this condition. GPCRs represent the largest superfamily of transmembrane proteins stimulated by numerous physiological and pathological processes. This series of processes triggers pathophysiological responses that could be related to the development of MetS diseases [19]. GPCRs play a role in the regulation of physiological processes and represent approximately 30% of the therapeutic targets that can be exploited [20,21]. Within these, it is worth highlighting the impact that this type of membrane receptor has on the treatment of pathologies with low-grade inflammation [22].

It has been observed that some GPCRs remain “orphan”, meaning that the endogenous ligands they interact with have not been identified. This area of study is fundamental to better understanding how GPCRs could modulate the development and progression of metabolic syndrome and other related diseases [23].

As defined in [24], several types of orphan GPCRs are divided mainly into the following categories: Class A (85 receptors), which have homology with hydrocarboxylic acids and lysophosphatidic acid receptors; Class B or Adhesion (33 receptors), such as known adhesion receptors; and Class C (8 receptors), which have homology with the *RA1G1* receptor that was identified as a retinoic acid-inducible gene.

This family of receptors has been a significant discovery in the field of disease research. Several researchers, including Sadat Alavi, have investigated orphan GPCRs with potential therapeutic applications for various central nervous system (CNS) disorders. A notable example is *GPR3*, which is highly expressed in the habenula region, a brain area implicated in the regulation of stress-related behaviors and neurological conditions like Alzheimer’s disease. Similarly, *GPR6* has been shown to promote neurite outgrowth in cultured cerebellar granule neurons, suggesting a role in neuronal development and repair. *GPR17*, another orphan GPCRs, is expressed in human adult neuroprogenitor cells and may contribute to neuronal regeneration. *GPR26* has been linked to the receptors for melatonin and sphingolipids, two classes of molecules with important roles in the CNS. Additionally, *GPR146* has been identified as a receptor that may be an effective treatment for diabetes-associated complications and may regulate the retinal pigment epithelium, which is disrupted in diabetic macular edema [25,26]. This study aims to determine the presence of the KLF14 and SREBF-1 transcription factor binding sites in the promoters of orphan receptor genes and to be able to associate some of these orphan receptors with the development of metabolic syndrome.

## 2. Results

### 2.1. Alignment Sequence Similarity-Based Process

Of the 126 orphan receptors, 85 belong to Class A, 33 to Class B or Adhesion, and 8 to Class C. Table 1 shows these results. Out of the 85 Class A receptors, 966 BS for KLF14 and SREBF-1 TFs were found. Of these BS, 501 were located on the positive (+) gene strand and 465 on the complementary strand (3′ to 5′). It was decided to exclude the BS on the complementary strand, resulting in 501 viable BS in the association process.

Similarly, of the 33 Class B or Adhesion receptors, 520 BS were found, of which 276 were located on the (+) strand and 244 on the complementary strand, for a total of 276 viable BS. For Class C, 8 ORs were analyzed, and 103 BS were found in the gene, of which 56 were located on the (+) strand and 47 were located on the complementary strand, for a total of 56 viable BS.

Furthermore, some important regions were identified in each of the genes of the orphan receptor promoters. In Class A, 2 BS were found in the silencer or inhibitory regions, as well as 64 BS in the enhancer regions. Additionally, 20 BS in the silencer regions and 34 in the enhancer regions were found, belonging to Class B or Adhesion genes receptors. Nevertheless, no sites were found in these important regions for Class C. Binding sites in the DR were also identified: 90 of them were from Class A, 49 from Class B or Adhesion, and 11 from Class C. These BS constitute a key factor for further analysis in the current study.

### 2.2. Important Region Identification and Similarity

An analysis was conducted regarding the incidence of ORs in the important regions. Of the 66 binding sites located in these regions in Class A, the *GPR15* receptor contains 1 of these binding sites; *GPR78* has 3 binding sites; *GPR148* has 4 binding sites; *GPR17*, *GPR19*, and *GPR50* have 9, 9, and 7 binding sites, respectively; *GPR3* contains 12 binding sites; and *GPR26* contains 22 binding sites. All this represents a high probability of incidence of these receptors with the KLF14 and SREBF-1 TFs for their possible involvement in metabolic syndrome.

In Class B or Adhesion, some receptors had a presence in these important regions, being allocated across the 54 identified binding sites: the *ADGRG6* receptor with 1 binding site in an important region; the *ADGRV1* receptor with 2 binding sites; the *ADGRG5*, *ADGRD2*, and *ADGRB1* receptors with 10, 9, and 7 binding sites, respectively; and the *ADGRA2* receptor with 14 binding sites. Despite this incidence in these Class A and B receptors, no binding sites of Class C receptors were identified in any important region of the gene.

The 1000 base pairs of nucleotides selected for the analysis were located upstream from the transcription start site. In this direction, regions such as the TATA box, the proximal elements region, and the distal elements region can be found, the latter being of greatest interest in the analysis performed. In this region, 90 binding sites of Class A, 49 of Class B or Adhesion, and 11 of class C were obtained, which constitute the fundamental object of analysis in the research. Binding sites are evident within this region. Notably, several orphan receptor genes exhibit multiple BS, including *GPR3*, *GPR17*, *GR176*, *P2RY10*, *GPR12*, *GPR85*, and, particularly, *GPR26* and *GPR37*. These findings, along with results from other studies, are encouraging and warrant further investigation.

Table 2 shows the results obtained for Class A, B or Adhesion, and Cass C receptors regarding their presence in the DR, respectively.

The BS located in the DR were identified as the most critical for our study and were, therefore, subjected to a more extensive analysis to ensure the accuracy and reliability of our results.

### 2.3. KLF14, SREBF-1, and oGPCRs Genic Expression Correlation

**Figure 1:**
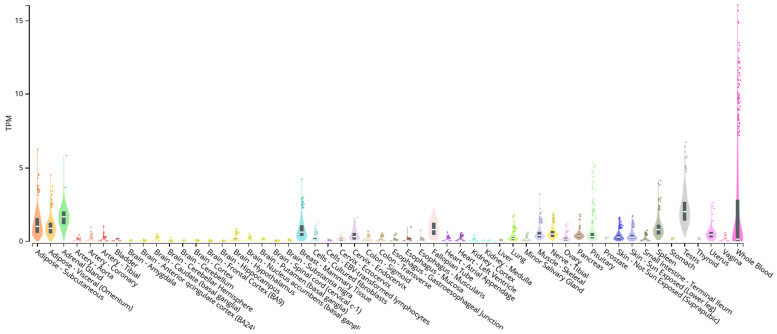
Bulk tissue gene expression for KLF14 (ENSG00000266265.4) [27]. TPM: transcripts per million. Colors within each distribution delineate the density of gene expression for the respective tissue. A higher concentration and intensity of color within the distribution signifies a greater proportion of samples exhibiting similar expression levels within that range.

An analysis has been conducted on the gene expression data of KLF14 and SREBF1, as well as information regarding the expression and function of the orphan receptors identified in our study. This analysis, performed using databases such as GTEx (Genotype-Tissue Expression), reveals tissue expression patterns relevant to metabolic syndrome. In the case of KLF14 (Figure 1), we observed expressions in metabolically relevant tissues, such as adipose tissue (both visceral omentum and subcutaneous). This expression suggests that KLF14 possesses the capacity to influence the expression of its target genes, including the orphan receptors, within these tissues.

**Figure 2:**
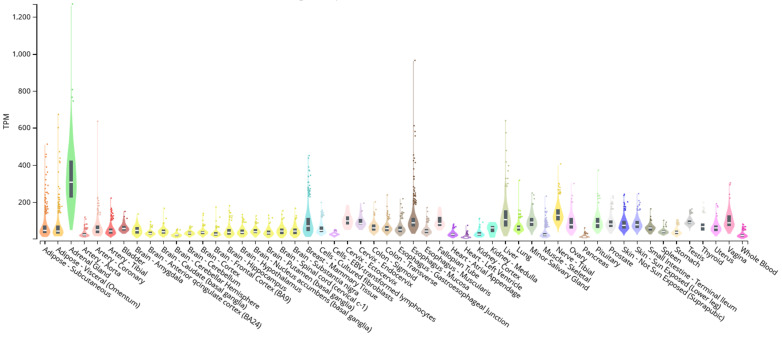
Bulk tissue gene expression for SREBF-1 (ENSG00000072310.18) [27]. TPM: transcripts per million. Colors within each distribution delineate the density of gene expression for the respective tissue. A higher concentration and intensity of color within the distribution signifies a greater proportion of samples exhibiting similar expression levels within that range.

Similarly, SREBF1 (Figure 2) exhibits broad and relatively high expression across a variety of tissues, including metabolically significant ones such as adipose tissue, liver, and muscle. The expression in the liver is particularly noteworthy, given SREBF1’s crucial role in regulating lipid and cholesterol metabolism within this organ.

To provide functional context, we considered the expression and known functions of the orphan receptors identified in our study. Several receptors demonstrate relevant functions in tissues where KLF14 and SREBF1 are expressed, suggesting a potential functional connection. For example, adipose tissue-related receptors like *GPR12* (associated with dyslipidemia and fat mass), *GPR21* (related to obesity resistance, inflammation, and insulin resistance), *GPR37*, and *GPR39* (both related to fat composition) have relevant functions in adipose tissue where both KLF14 and SREBF1 are expressed; liver function-related receptors like *GPR21* and *GPR132* (associated with hepatobiliary homeostasis) have important functions in the liver where SREBF1 exhibits notable expression; a vascular function-related receptor like *MAS1* (related to endothelial function) has important functions in coronary arteries where SREBF1 is expressed; and immune function-related receptors like *GPR84* and *GPR183* (both related to immune functions) have relevant functions in blood where KLF14 shows high expression. These orphan receptors, along with others identified in our analysis, have relevant functions in metabolism, energy homeostasis, and inflammation—processes that are fundamental in metabolic syndrome. The observation that KLF14 and SREBF1 are expressed in tissues where orphan receptors have relevant functions suggests a possible functional link. While our study predicts the binding of these transcription factors to regulatory regions near these receptors, the expression of the transcription factors in the same tissues supports the biological plausibility that this regulation is occurring. We acknowledge that these gene expression data provide correlational evidence. While the expression of transcription factors and orphan receptors in the same tissues supports the possibility of functional regulation, further studies are required to directly demonstrate the regulation of orphan receptor expression by KLF14 and SREBF1. These studies could include gene expression analyses following the manipulation of KLF14 or SREBF1 expression or reporter assays to evaluate the activity of orphan receptor promoters in the presence of these transcription factors. In conclusion, the incorporation of gene expression data for KLF14 and SREBF1, along with information on the expression and function of orphan receptors, provides additional evidence supporting the functional consequences of our findings. These data suggest that the transcription factors we studied have the capacity to influence the expression of genes relevant to metabolic syndrome, including the orphan receptors identified in our analysis.

### 2.4. Identification of GPCRs Associated with Metabolic Syndrome (GPCRs-MetS)

Using the eukaryotic promoter database, we identified putative binding sites for the transcription factors KLF14 and SREBF-1 in the promoters of a panel of GPCRs associated with metabolic syndrome. Most of the analyzed GPCRs exhibited multiple putative binding sites for KLF14 and SREBF-1, especially when considering a *p*-value of 0.01. A significant proportion of binding sites were in the distal region of the promoters, suggesting a potential regulatory role for these transcription factors in GPCRs expression.

The distribution and number of BS varied considerably among the different receptors, indicating differential regulation by KLF14 and SREBF-1. These results suggest that KLF14 and SREBF-1 may play a significant role in the transcriptional regulation of a subset of GPCRs associated with MetS. The presence of multiple BS in the distal promoter region indicates that these transcription factors could modulate basal expression of these genes and respond to specific stimuli.

No BS for either KLF14 or SREBF1 was identified in the promoters of *MC4R*, *5-HT2C*, *MC3R*, *S1PR2*, *FFAR1/GPR40*, and *HCR2* within this database, despite their known association with metabolic syndrome [28,29,30,31,32,33,34,35]. The results are summarized in Table 3.

After an exhaustive review of the literature, GPCRs associated with metabolic syndrome were identified. The promoters of the genes encoding these receptors were extracted from the GenBank-NCBI (National Center for Biotechnology Information) database according to the established inclusion criteria (1000 base pairs upstream of the transcription start site).

Table 4 shows the number of binding sites for the KLF14 and SREBF-1 TFs, respectively, found in the genes of these MetS-associated receptors. The number of BS in both the positive and complementary strands are shown, with viable results being those BS located in the (+) strand. Additionally, BS present in important regions (enhancers or silencers) within the receptor gene were identified, as well as those found near or within the DR in the elements upstream of the transcription start site. Based on these results, it was essential to continue the analysis with those BS present in the DR of the gene for subsequent analysis and comparison with the results obtained in the analysis of orphan receptors.

The relevance of the following receptors can be observed: leptin receptor (*LEPR*), adiponectin receptor (*ADIPOR1*), ghrelin receptor (*GHSR*), serotonin 5-*HT2C* receptor, cannabinoid receptor *CB1/CNR1*, orexin receptor *OX1R/HCRTR1*, and sphingosine-1-phosphate receptor *S1PR1*. All of these were found to have a KLF14 transcription factor BS in the DR, except for *OX1R/HCRTR1* and *S1PR1*, which had 8 and 11 BS in this region, respectively.

Regarding the analysis performed for the transcription factor SREBF-1, the following was obtained: both the leptin receptor (*LEPR*) and the angiotensin II receptor (*AGTR1*), as well as the cannabinoid receptor *CB1/CNR1* and the sphingosine-1-phosphate receptor *S1PR1*, were found to contain a BS in the DR. Although this may represent a much lower percentage of presence, this result, together with the previous one, allows us to draw strong conclusions regarding the association that these GPCRs may have with the orphan GPCRs and their relationship with MetS.

### 2.5. Clustering Analysis for the Bioinformatics Outcomes

A clustering analysis was conducted to identify groups of orphan GPCRs and GPCRs-MetS promoters that exhibit similar binding patterns for KLF14 and SREBF-1. Six variables were employed: the number of KLF14 and SREBF-1 BS in the DR, the number of KLF14 and SREBF-1 BS in the gene’s (+) strand, and KLF14 and SREBF-1 similarity.

**Figure A1:**
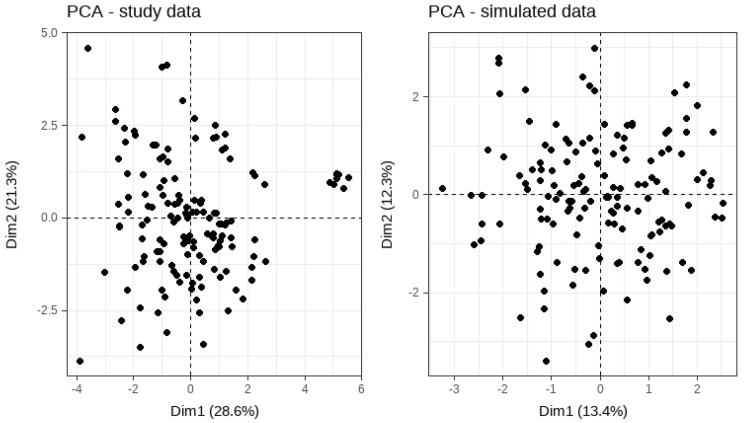
A plot to check possible groups. Dim1: Dimension 1. Dim2: Dimension 2.

Before clustering, it was essential to determine if genuine clusters existed within the dataset. To illustrate the importance of this preliminary analysis, clustering was applied to two datasets: one containing genuine groups (study data) and the other randomly simulated dataset without such groups. Figure A1 confirmed the presence of genuine clusters in the study dataset, while the simulated data did not exhibit any clear grouping.

**Figure A2:**
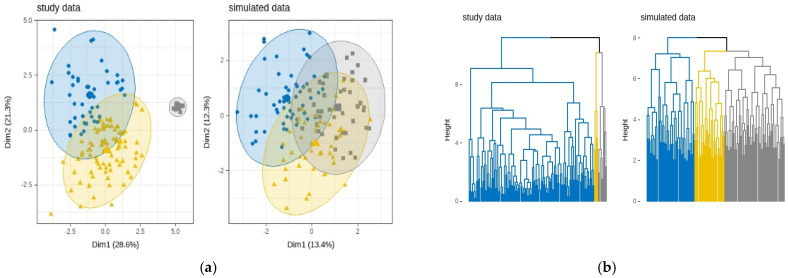
Clustering to visualize group formation. (**a**) K-means. (**b**) Hierarchical. Colors represent different clusters, and symbols represent sub-groups with the same features within each cluster.

In the study dataset, a slight separation among observations can be observed, suggesting the potential formation of clusters. In contrast, the simulated dataset exhibits more compact and dispersed observations, making it more difficult to discern distinct groups. When applying clustering to both datasets (Figure A2a,b), the formation of groups becomes slightly more evident.

**Figure 3:**
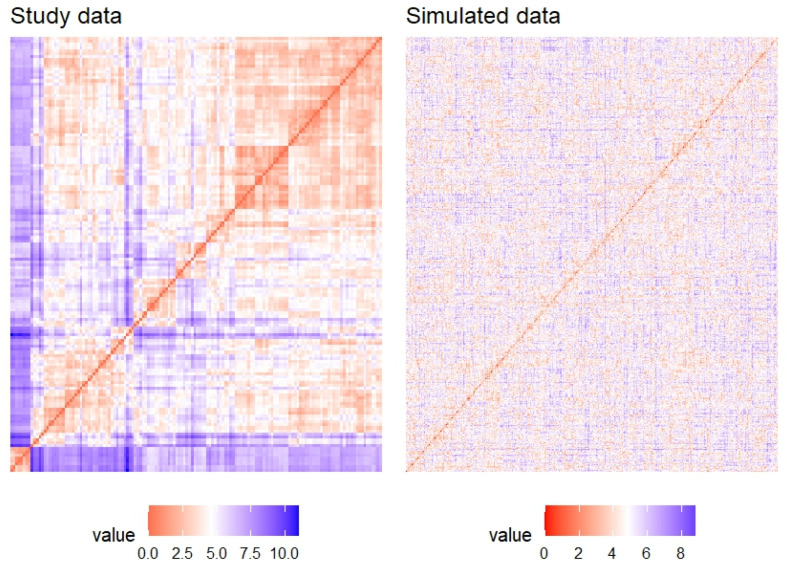
A VAT plot to confirm group formation in the data.

Finally, the Visual Assessment of Cluster Tendency (VAT) was used to visually assess (Figure 3) whether the data tended to form groups. Unlike the simulated data, the study dataset demonstrates the existence of clusters, suggesting that various clustering algorithms could be employed to identify patterns in the distribution of KLF14 and SREBF-1 BS within the promoters of GPCRs and orphan GPCRs genes. This analysis would provide insights into the potential regulatory roles of these TFs in the expression of these receptors.

#### 2.5.1. PCA for the Distribution of Individual Observations 

**Figure 4:**
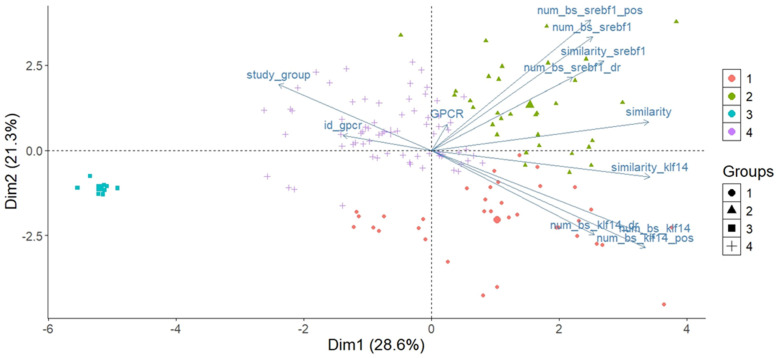
A PCA plot with observations and variables. Dim1: Dimension 1. Dim2: Dimension 2.

**Figure A3:**
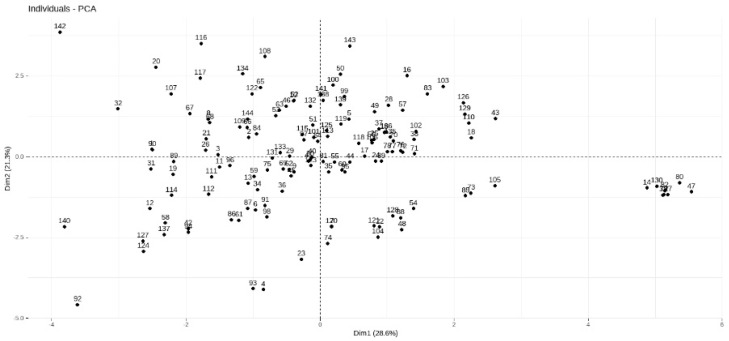
A PCA plot of the individual observation’s distribution. Dim1: Dimension 1. Dim2: Dimension 2. Numbers represent GPCRs and GPCRs-MetS genes coded in the database.

Initial two-dimensional PCA (Figure A3 and Figure 4) revealed a highly skewed distribution of encoded receptors, with Dimension1 (Dim1) accounting for nearly all variability. Subsequent k-means clustering identified four distinct gene groups based on the density of KLF14 and SREBF-1 BS. A biplot demarcated these groups, indicating diverse transcriptional regulatory profiles. Notably, the density of KLF14 BS in the DR and overall similarity were key factors in cluster formation. Group 1 exhibited a high density of distal KLF14 BS and low similarity, suggesting tighter KLF14-mediated regulation. Conversely, Group 3 showed lower distal KLF14 binding density and higher similarity. Furthermore, the variable representing the study group moderately modulated clustering, hinting at dataset-specific binding profiles. These findings underscore the heterogeneity of KLF14 and SREBF1 binding patterns, implying intricate transcriptional regulation mediated by these factors.

#### 2.5.2. K-Means Clustering Algorithm

**Figure A4:**
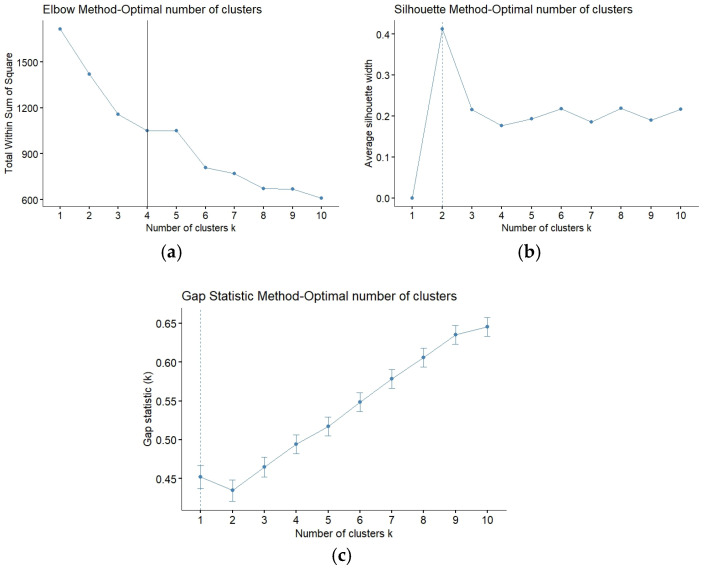
Optimal number of clusters. (**a**) Elbow method. (**b**) Silhouette method. (**c**) Gap statistic method.

The elbow method was employed to apply the K-means algorithm. As shown in Figure A4a, the ‘elbow’ point was identified around K = 4. This suggests that a clustering model with four clusters represents a suitable offset between minimizing within-cluster variance and maintaining a manageable number of groups.

Figure A4b presents the silhouette plot. The peak in this plot indicates that the data are optimally divided into two clusters as the average silhouette width reaches its maximum value at K = 2. This suggests that the data points are more compact within their respective clusters and well separated from other clusters. Therefore, based on the silhouette method, the optimal number of clusters for this dataset is two. Figure A4c shows the gap statistic plot. Unlike the previous methods, the gap statistics tend to increase with the number of clusters, and the error bars largely overlap. This suggests that the gap statistic method is unable to determine an optimal number of clusters for this dataset, possibly due to the variability of the data or the limitations of the method itself.

The complete method indicates that the Dindex value, a metric used to assess clustering quality, decreases as the number of clusters increases, as expected. It is important to note that this method relies on a series of indices to evaluate the data and determine the optimal number of clusters. Among all indexes:

- Five proposed two as the best number of clusters;
- Four proposed three as the best number of clusters;
- Two proposed four as the best number of clusters;
- One proposed five as the best number of clusters;
- Two proposed six as the best number of clusters;
- Three proposed eight as the best number of clusters;
- Five proposed nine as the best number of clusters;
- Five proposed ten as the best number of clusters.

So, according to the majority rule of this method, the best number of clusters is two.

Different values of K (2–7) were evaluated for the k-means clustering with the Euclidean distance, and it was observed that as K increased, the within-cluster sum of squares (WBSS) consistently decreased, indicating that data points within each cluster became increasingly similar. However, the rate of decrease in WBSS diminished as more clusters were added, suggesting that the inflexion point (or ‘elbow’) in the numerical results analysis could be located at K = 6.

Finally, a cluster stability analysis was conducted using the k-means method and 100 resampling iterations to assess the robustness of the obtained clusters for different values of K. The Jaccard index, recovery rate, and dissolution rate of the clusters were calculated to evaluate their consistency. A general trend towards lower cluster stability was observed as the value of K increased, as indicated by decreasing Jaccard index values and higher dissolution rates. This suggests that when dividing the data into a larger number of groups, these clusters tend to be less well-defined and more susceptible to variations in the sample. Additionally, clusters with varying levels of stability were identified, indicating that some groupings were more robust and representative than others. Considering the Jaccard index values, the optimal number of clusters seems to be four (0.89, 0.78, 0.89, 0.77), demonstrating good stability in the data.

Table A1 summarizes the proposed optimal number of clusters for the analyzed dataset, as determined by each method.

Considering the characteristics of each method, the optimal number of clusters for the dataset was determined to be four, based on the accuracy of the elbow method and the cluster stability evaluation provided by Clusterboot.

To further investigate the impact of KLF14 and SREBF-1 on orphan GPCR and GPCR genes, a thorough analysis was conducted using K-means clustering and principal components analysis (PCA) with K = 4. The first two main components explained a substantial portion of the variance in the data. The first main component, accounting for 28.62% of the variance, was strongly associated with variables related to the expression of KLF14 and SREBF-1 genes, suggesting that differences in the expression of these genes are a major source of variation among samples. The second main component, explaining 21.27% of the variance, was also related to the expression of these genes, as well as the variable related to the study group.

**Figure 5:**
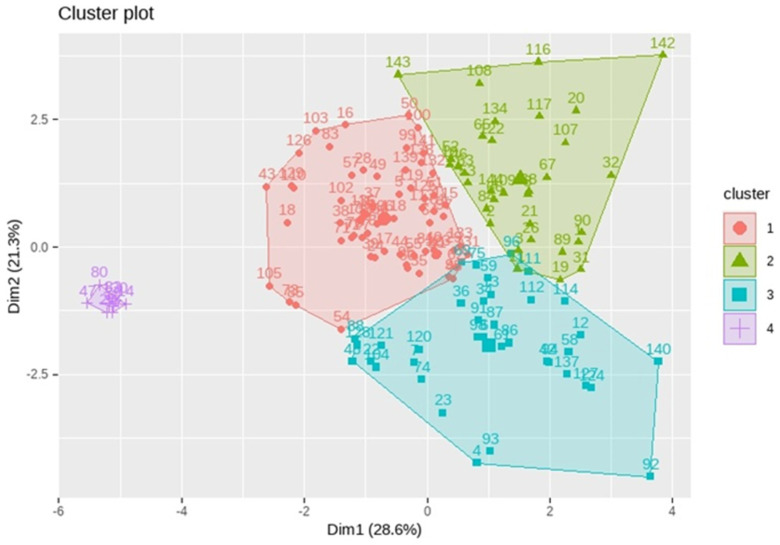
K-means cluster plots. Separated group’s view. Dim1: Dimension 1. Dim2: Dimension 2. Colors represent different clusters; numbers represent GPCRs and GPCRs-MetS genes, and symbols represent subgroups within each cluster.

Figure 5 shows the distribution of observations by groups after applying the K-means algorithm. Each point represents an observation, and different colors and shapes indicate membership in each of the four identified clusters.

**Figure 6:**
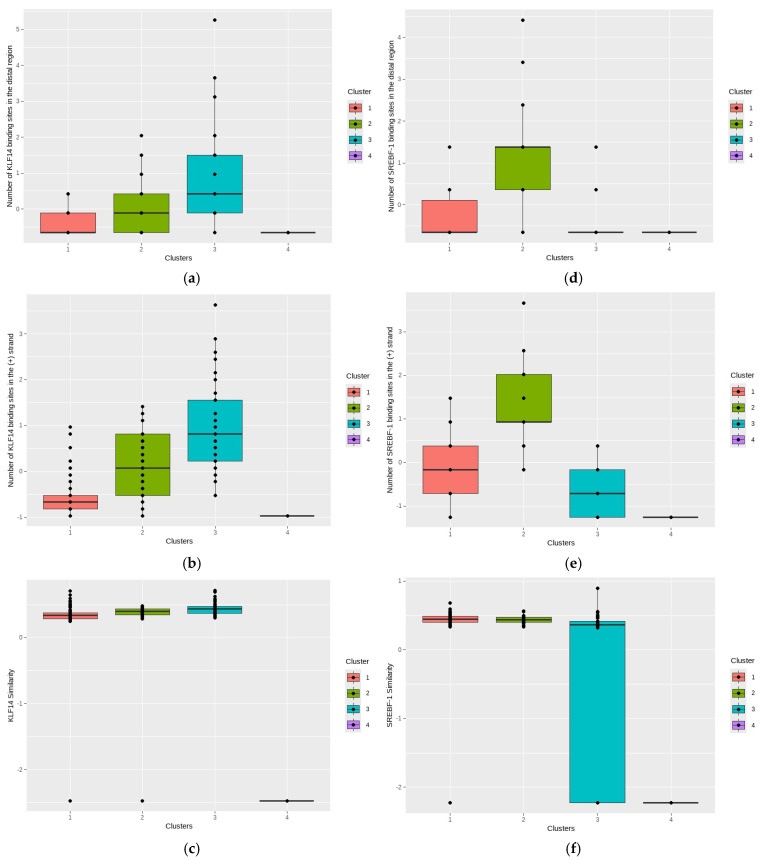
Cluster box plots: (**a**) variable number of KLF14 binding sites in the distal region; (**b**) variable number of KLF14 binding sites in the positive strand; (**c**) variable KLF14 similarity; (**d**) variable number of SREBF-1 binding sites in the distal region; (**e**) variable number of SREBF-1 binding sites in the positive strand; (**f**) variable SREBF-1 similarity.

The obtained clusters exhibit clear separation, with Cluster 1 (blue circles) being well separated and compact. Clusters 2, 3, and 4 (yellow triangles, grey squares, and red crosses) are closer and have more elongated shapes. To better understand the variables influencing cluster formation, box plots are shown in Figure 6a–f, illustrating their distribution in each cluster.

Box plot analysis revealed significant differences in the number of BS in the DR among the four identified clusters (Figure 6a). Cluster 1 exhibited the lowest median and variability in the number of KLF14 binding sites, with the median approximating zero. Cluster 2 demonstrated a slight increase in both median and variability compared to Cluster 1, with the presence of some outliers. In contrast, Cluster 3 presented the highest median and variability in the number of KLF14 binding sites, characterized by multiple high outlier values. Cluster 4 displayed the lowest distribution and variability across all clusters, with most values clustering near zero. These observed differences in the distribution of KLF14 binding sites suggest a potential differentiation of the clusters based on KLF14 regulation. Cluster 3 is characterized by a greater quantity of KLF14 binding sites and increased variability, which may reflect heightened KLF14 activity or regulation within this group. Conversely, Clusters 1 and 4 exhibit a diminished quantity of KLF14 binding sites, potentially indicating a reduced influence of KLF14 in gene regulation within these groups. The amount of BS for KLF14 in the (+) strand of the receptor gene varied significantly among clusters (Figure 6b), suggesting differences in the transcriptional regulation of these genes. Cluster 1 exhibited the lowest median and variability in KLF14 binding site numbers. Cluster 2 demonstrated a marginal increase in median and variability compared to Cluster 1, with the presence of some outliers. In contrast, Cluster 3 presented the highest median and variability in KLF14 binding site numbers, characterized by multiple high outlier values. Cluster 4 displayed the lowest distribution and variability across all clusters, with most values clustering near zero. These differences in the distribution of KLF14 binding sites suggest a potential differentiation of the clusters based on KLF14 regulation. Cluster 3 is characterized by a greater number of KLF14 binding sites and increased variability, which may reflect heightened KLF14 activity or regulation within this group. Analysis of the similarity score (Figure 6c) between KLF14 BS and receptor genes revealed a high degree of similarity in KLF14 binding site patterns for Clusters 1, 2, and 3, as evidenced by the consistent distribution and median values observed across these groups. Clusters 1, 2, and 3 exhibited a narrow distribution of KLF14 similarity scores, with medians clustering around a similar value. The variability within each of these clusters was also low. In stark contrast, Cluster 4 displayed a distinctly different pattern of KLF14 similarity. The similarity scores for Cluster 4 were markedly lower and less variable compared to Clusters 1, 2, and 3, with most values clustering near a lower value. These findings suggest a high level of conservation in KLF14 binding site patterns among Clusters 1, 2, and 3. The divergent pattern observed in Cluster 4 indicates a potential shift in KLF14 binding site similarity within this group, suggesting a distinct regulatory context or functional divergence compared to the other clusters.

The number of SREBF-1 BS in the DR (Figure 6d) was associated with the distribution of orphan GPCRs and GPCRs across clusters. The analysis revealed variability in SREBF-1 binding site counts among the clusters. Cluster 1 exhibited a low median and limited variability in SREBF-1 binding site numbers, with a median clustering near zero. Cluster 2 demonstrated an increase in median and variability compared to Cluster 1, with the presence of outliers. In contrast, Clusters 3 and 4 displayed the lowest distribution and variability, with most values clustering near zero. These differences in the distribution of SREBF-1 binding sites suggest a potential differentiation of the clusters based on SREBF-1 regulation. Cluster 2 is characterized by a greater number of SREBF-1 binding sites and increased variability, which may reflect heightened SREBF-1 activity or regulation within this group. Conversely, Clusters 1, 3 and 4 exhibit a diminished number of SREBF-1 binding sites, potentially indicating a reduced influence of SREBF-1 in gene regulation within these groups.

The distribution of SREBF1 BS in the (+) strand (Figure 6e) of the receptor gene varied significantly among clusters, suggesting differences in SREBF1-mediated transcriptional regulation. Cluster 1 exhibited a low median and limited variability in SREBF-1 binding site numbers. Cluster 2 demonstrated an increase in median and variability compared to Cluster 1, with the presence of outliers. In contrast, Clusters 3 and 4 displayed the lowest distribution and variability, with most values clustering near zero. These differences in the distribution of SREBF-1 binding sites suggest a potential differentiation of the clusters based on SREBF-1 regulation. Cluster 2 is characterized by a greater number of SREBF-1 binding sites and increased variability, which may reflect heightened SREBF-1 activity or regulation within this group. Conversely, Clusters 1, 3 and 4 exhibit a diminished number of SREBF-1 binding sites, potentially indicating a reduced influence of SREBF-1 in gene regulation within these groups.

Analysis of (Figure 6f) between SREBF1 BS and receptors revealed distinct differences in SREBF-1 similarity between Cluster 3 and the other clusters. Clusters 1, 2, and 4 exhibited low and consistent SREBF-1 similarity, with medians clustering around a similar value. In contrast, Cluster 3 displayed significantly higher SREBF-1 similarity. These findings suggest low conservation of SREBF-1 binding site patterns among Clusters 1, 2, and 4, while Cluster 3 exhibits a divergent, higher SREBF-1 similarity pattern.

Overall, this analysis revealed a diverse distribution of BS for the TF KLF14 and SREBF1 across the different receptor clusters. Cluster 3 had the highest density and similarity of BS for both factors, suggesting a greater potential for regulating target genes in this group.

### 2.6. Regression Analysis for Orphan GPCRs and KLF14 and SREBF-1

A Poisson model was suitable for the relationship between KLF14 BS in the DR and the (+) strand. Nevertheless, the overdispersion test applied indicated significant overdispersion for KLF14 (deviance ratio = 1.75, *p* < 0.001), suggesting unobserved heterogeneity or a more complex counting process.

In contrast to KLF14, the SREBF-1 model exhibited no evidence of overdispersion, as indicated by a residual deviance to degrees of freedom ratio of 1.0096 (*p* = 0.452). Therefore, the Poisson model was sufficient.

#### 2.6.1. Negative Binomial Regression Model for KLF14

Consequently, a negative binomial model was fitted for KLF14 to account for overdispersion. The results (Table 5) show that the number of KLF14 BS in the (+) strand was significantly associated with the number of BS in the DR (coefficient = 0.10986, *p* < 0.001). Each additional BS in the (+) strand was associated with an 11.6% increase in the number of BS in the DR (95% CI: 0.0776, 0.1443). The overdispersion parameter (θ = 1.274) confirmed the presence of overdispersion, supporting the use of a negative binomial model.

The negative binomial model provided a better fit to the data, as indicated by its lower residual deviance (136.71) compared to the Poisson model. The AIC (397.39) further supports this finding.

The model was also fitted with another predictor variable to check its contribution over the response variable. The results revealed a significant positive association between the two variables [coefficient = 10.831 (95% CI: 4.1175–20.2363), *p* = 0.00951], indicating that as KLF14 similarity increases, so does the number of KLF14 BS in the DR. These findings support the hypothesis that there is a direct link between these two variables, suggesting that KLF14 similarity may be a key determinant of KLF14 BS in the DR levels. The dispersion parameter (0.9107) confirms the overdispersion in the data, and the constrained residual deviance in this model suggests a more accurate representation of the underlying relationship between the variables, leading to improved predictive performance.

The model was extended to include both predictors identified through bioinformatics analysis. The results of a negative binomial regression with KLF14 BS in the (+) strand and KLF14 similarity as predictors to investigate factors influencing the number of KLF14 BS in the DR reveal that both the amount of KLF14 BS in the (+) strand (coefficient = 0.08269, *p* < 0.001) and KLF14 similarity (coefficient = 4.28924, *p* = 0.02437) were significantly associated with an increase in the number of BS in the DR of the gene. For each unit increase in KLF14 similarity, an increase of exp (4.28924) ≈ 73.7% in the number of BS was observed, suggesting a more pronounced effect of this predictor. The dispersion parameter Theta (θ = 1.4771) confirmed the presence of overdispersion in the data. The residual deviance of the negative binomial model (128.27) was lower than that of the previous model with a single predictor, suggesting a better fit to the data. The BIC (Bayesian Information Criterion) also revealed this conclusion of goodness of fit [BIC (modelbn1) = 413.3846, BIC (modelbn2) = 395.9584] and BIC (modelbn3) = 395.9584, indicating a better balance of Model 2 and 3 between fit and complexity and suggesting that the two predictors model is a better option to represent the data.

**Figure A7:**
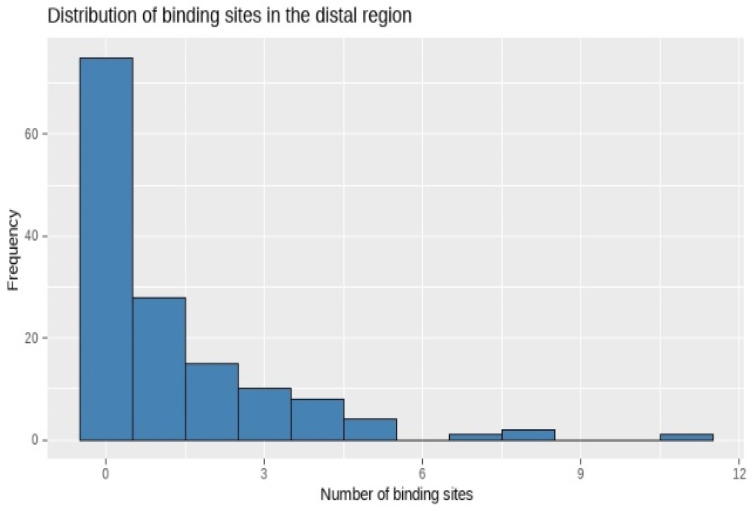
A histogram of the response variable.

**Figure A8:**
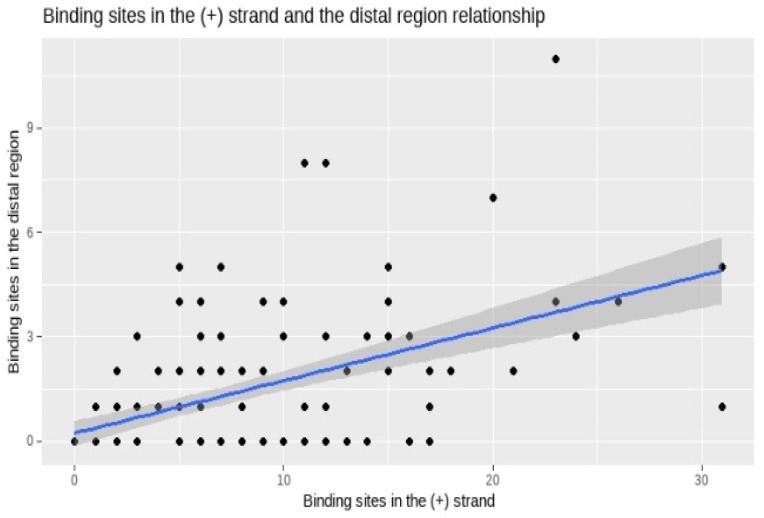
A scatter plot. The relationship between variables. Black points represent individual data; blue line indicates the regression line; the gray area shows the confidence interval.

Figure A7 displays the histogram of the response variable, visually confirming its count-based nature. The distribution is positively skewed, with most observations concentrated in the lower values of the BS count. The mode falls within the first interval (0–3). Figure A8 shows a positive relationship between the variables, with a regression line indicating that as the number of BS in the (+) strand and the KLF14 similarity increases, so does the number of BS in the DR. However, the relationship is not perfect, and there is some variability in the data. The confidence bands around the regression line show that the relationship is more certain in some regions than in others. Overall, the results support a positive association between the two variables.

**Figure A9:**
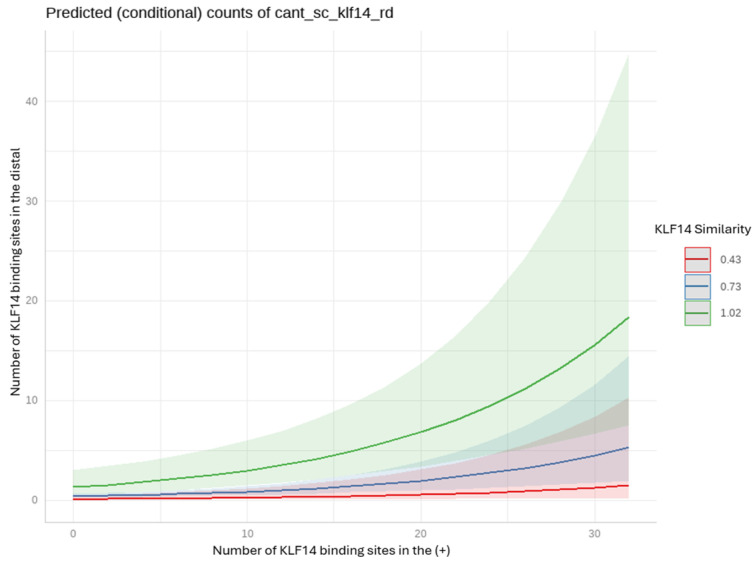
A marginal effects plot for the negative binomial model.

A marginal effects plot was created to visualize the relationship between the predictors and the response variable (Figure A9). The plot shows a positive association between the number of KLF14 BS in the (+) strand and the total number of BS in the DR, as expected based on our previous findings. This indicates that an increase in KLF14 BS in the (+) strand is associated with an increase in the total number of BS in the DR, even when controlling for other variables.

The marginal effects plot shows that the relationship between the amount of KLF14 BS in the (+) strand and the total number of BS in the DR is moderated by similarity. As similarity increases, the positive association between these two variables becomes stronger. The non-linearity of the lines suggests a more complex relationship than a simple linear one. Based on these findings, we can conclude that the number of KLF14 BS in the (+) strand is a significant predictor of the total number of BS in the DR, and the effect of the number of KLF14 BS in the (+) strand is amplified by increasing levels of similarity.

#### 2.6.2. Poisson Regression Model for SREBF-1

A Poisson regression model was fitted to investigate the factors associated with SREBF-1 binding in the regulatory region, including the amount of SREBF-1 BS in the (+) strand and sequence similarity as predictors. The results (Table 6) revealed a positive and statistically significant association (coefficient = 0.351, 95% CI: 0.262, 0.440, *p* < 0.001). This indicates that for every one-unit increase in the number of BS in the (+) strand, we can expect an increase in the number of BS of SREBF-1 in the DR, on average. These findings highlight the importance of the amount of SREBF-1 BS in the (+) strand as a key determinant of SREBF-1 expression in the DR.

Likewise, the same regression model was fitted with the SREBF-1 similarity obtained in the previous analysis. This predictor presents a larger coefficient and a wider confidence interval (coefficient = 4.738, 95% CI: [2.248, 9.45], *p* < 0.001), suggesting a stronger but less precise association compared to the number of BS in the (+) strand. This result, together with the previous analysis that showed a positive effect, suggests that both the similarity and the number of BS of SREBF1 contribute independently to explain the variability in the count of SREBF1. However, the effect size and precision of the estimate are greater for the SREBF-1 similarity.

Finally, the Poisson regression model was fitted to assess the combined effect of the number of BS in the (+) strand and the SREBF-1 similarity on the number of BS in the DR. Results showed that both predictors have a positive and statistically significant effect on the response variable. Specifically, for every one-unit increase in the number of the SREBF-1 BS in the (+) strand, we expect an average increase of exp (0.2995) = 1.34 = 34% in the number of BS in the DR, holding the similarity constant (95% CI: 1.22–1.43). Similarly, a one-unit increase in SREBF-1 similarity is associated with an average increase of 51.91% in the number of BS in the DR, holding the other variable constant (95% CI: 3.07–59). These results suggest that both the number of BS and the similarity of SREBF1 play an important role in determining the final count.

When comparing this model to previous models that included only one of the predictors, the current model presents a lower Bayesian Information Criterion (BIC) value (280.29), indicating a better fit to the data. These results suggest that both predictors, the number of BS in the (+) strand and the similarity of SREBF-1, provide valuable and complementary information to explain the variability in the number of BS in the DR of SREBF-1.

**Figure A10:**
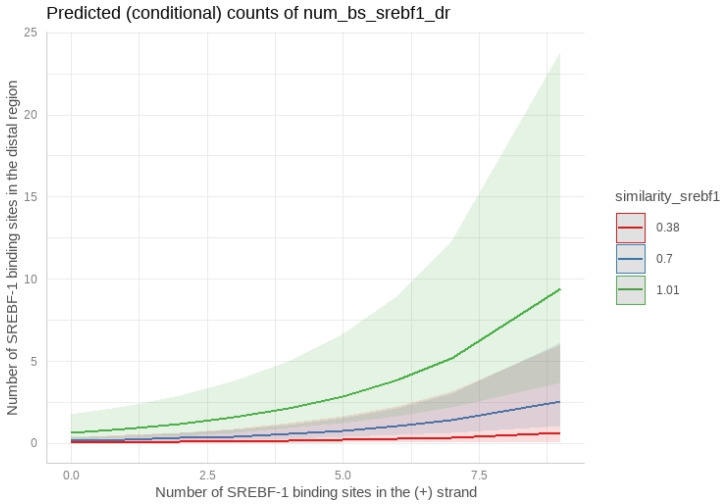
A marginal effects plot for the Poisson model.

To visualize the impact of predictors on the response variable, Figure A10 presents marginal effects plots, illustrating how changes in one predictor modulate the expected count of BS in the DR, holding other predictors constant.

Marginal effects analysis demonstrated a non-linear positive association between the number of SREBF-1 BS in the (+) strand and the DR, particularly in regions with higher sequence similarity. This suggests a synergistic interaction between these two factors. Our findings support the hypothesis that SREBF-1 binding sites in the DR is modulated by both the density of BS and the local genomic context.

### 2.7. Comparison Between Receptor Groups

A negative binomial regression model was fitted to examine the association between BS density in the DR and predictor variables, accounting for receptor class [orphan GPCR vs. GPCR-MetS] in the context of KLF14. The model included previously examined variables and an interaction term between BS in the (+) strand density, the similarity, and the receptor class. The results (Table 7) reveal that KLF14 BS density in the (+) strand and KLF14 similarity significantly modulated the DR’s total number of BS. However, we found no evidence of differential effects between orphan GPCR and GPCR-MetS receptor groups. Specifically, increases in (+) strand BS density and KLF14 similarity were associated with significant increases in total BS density (coefficients: 0.0744, *p* < 0.001; 4.2839, *p* = 0.0235, respectively). The class indicator and the interaction term were non-significant (*p* = 0.3579 and 0.1910, respectively), suggesting that the relationship between the (+) strand BS density and total BS density was similar across both receptor groups.

**Figure 7:**
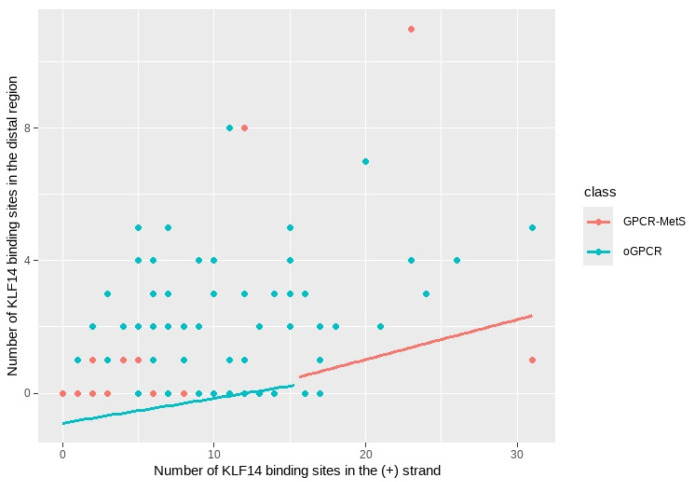
A scatter plot with fitted lines from a negative binomial regression model comparing receptor groups. GPCRs-MetS: G protein-coupled receptors associated with metabolic syndrome. oGPCRs: orphan G protein-coupled receptors.

Figure 7 illustrates these findings with a scatter plot displaying fitted lines for each receptor group. While a positive correlation between the variables was observed, the fitted lines for GPCRs-SM and oGPCRs groups exhibited slightly different slopes, suggesting a potential interaction. This indicates that increases in KLF14 BS density in the (+) strand might have a stronger effect on total BS density in the GPCRs-SM group compared to the oGPCRs group. That is why our statistical analysis did not confirm a significant difference between these groups.

Similarly, a Poisson regression model was fitted to examine the relationship between SREBF-1 BS density in the RD and predictor variables, accounting for the receptor class. The model (Table 8) revealed that SREBF-1 BS density in the (+) strand significantly modulated total BS density. However, there was no evidence of differential effects between orphan GPCR and GPCR-MetS groups. Specifically, increases in (+) strand BS density were associated with significant increases in total BS density (coefficient = 0.28852, *p* < 0.001).

Nevertheless, the class indicator coefficient (GPCR-MetS) was non-significant (*p* = 0.258), suggesting no substantial interception difference between receptor groups. Moreover, the interaction term was also non-significant (*p* = 0.421), indicating that the relationship between (+) strand BS density and total BS density was similar across both receptor groups.

**Figure 8:**
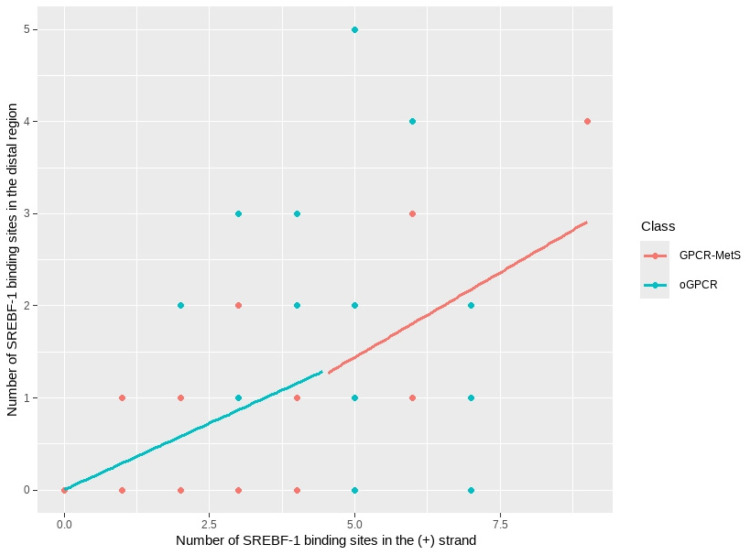
A scatter plot with fitted lines from a Poisson regression model comparing receptor groups. GPCR-MetS: G protein-coupled receptors associated with metabolic syndrome. oGPCR: orphan G protein-coupled receptors.

Figure 8 illustrates these findings with a scatter plot displaying fitted lines for each receptor group.

As observed with KLF14, a positive correlation between SREBF-1 BS density in the (+) strand and total BS density in the DR was evident. This suggests that the presence of (+) strand BS might facilitate SREBF-1 binding in the DR despite its low expression levels in receptor promoters. Although both receptor classes exhibited a positive relationship, the slightly steeper slope for the GPCR-MetS class hints at a potentially stronger association. This could indicate subtle differences in SREBF-1 binding mechanisms between classes. Overall, (+) strand SREBF-1 BS density serves as a robust predictor of total BS density in both orphan GPCR and GPCR-MetS groups.

## 3. Discussion

The bioinformatics analysis identified a potential interaction network among orphan GPCR, KLF14, and SREBF-1 in the context of MetS. This finding aligns with previous research demonstrating the expression of certain orphan receptors, such as *GPR26* (decreased in the heart and aorta) and *GPR39* (isoforms vary by tissue and MetS model), in tissues germane to MetS [36].

### 3.1. Orphan Receptors Analysis

Our bioinformatics analysis identified significant enrichment of binding sites for KLF14 and SREBF-1 within the promoter regions of orphan GPCRs, suggesting a potential regulatory role for these transcription factors in the expression of these receptors. Some of them, including *GPR26*, *GPR37*, and *GPR85*, exhibited a high frequency of binding sites for KLF14 and SREBF-1, suggesting their potential involvement in the molecular pathways underlying MetS. This aligns with previous research demonstrating the involvement of orphan GPCR in energy metabolism and diabetes.

First, our analysis of the tissue expression patterns of KLF14, SREBF1, and orphan receptors reveals a significant overlap in tissues relevant to metabolic syndrome, suggesting a potential functional interaction between these transcription factors and the receptors. Then, KLF14 demonstrates high expression in adipose tissue, the adrenal gland, mammary tissue, fallopian tubes, skeletal muscle, the pituitary gland, and whole blood. SREBF1 exhibits notable expressions in subcutaneous and visceral adipose tissue, the adrenal gland, coronary arteries, mammary tissue, esophageal mucosa, liver, and pituitary gland [27]. This expression of both transcription factors in adipose tissue is particularly salient as receptors such as *GPR12* [37], *GPR21* [38,39], *GPR37* [40,41], and *GPR39* [42], which have significant roles in lipid metabolism and adipose tissue regulation, could also be regulated by these transcription factors in this tissue. Furthermore, the expression of SREBF1 in the liver, coupled with the hepatic function of receptors like *GPR21* [39] and *GPR132* [43], implies a potential regulation of hepatobiliary homeostasis by SREBF1. The expression of KLF14 in blood, in conjunction with the immune functions of receptors like *GPR84* [44] and *GPR183* [45], raises the possibility that KLF14 may regulate these receptors in immune cells. Collectively, these tissue expression patterns lend credence to the hypothesis that KLF14 and SREBF1 can regulate the expression of orphan receptors in tissues crucial for metabolic syndrome.

On the other hand, *GPR50* has been shown to play a role in the regulation of energy metabolism and obesity [46]. Similarly, *GPR83* has been implicated in the central regulation of energy metabolism through its modulation of the hypothalamus–pituitary–adrenal axis [47].

Even when in this study, the *GPR55* only showed the presence of just two binding sites in the distal region, recent studies have also highlighted the potential role of this receptor in weight management. While some evidence suggests that *GPR55* activation may lead to decreased food intake and weight loss, other studies have shown an association between *GPR55* activation and increased adiposity [48].

Aligned with our findings, *GPR21*, which also had binding sites associated with the transcription factors, is expressively associated with developing type 2 diabetes, and it is a potential therapeutic target for its treatment [49].

While we observe an overlap in the expression of KLF14, SREBF1, and orphan receptors in tissues relevant to metabolic syndrome, specific expression patterns are also noted, which may have functional implications. For instance, the notable expression of SREBF1 in the liver, a central organ in lipid metabolism, suggests a particularly significant role for SREBF1 in regulating lipid metabolism-related gene expression within this tissue. This could include the regulation of orphan receptors with hepatic functions, such as *GPR132* [43], which is associated with hepatobiliary homeostasis. Conversely, the high expression of KLF14 in whole blood suggests a potential critical function in immune cells. This may imply the regulation of orphan receptor expression with immune functions, such as *GPR84* [44] and *GPR183* [50], which are involved in T-cell and dendritic cell function, respectively. These specific tissue expression patterns suggest that KLF14 and SREBF1 may exert distinct regulatory functions in different tissues and that the regulation of orphan receptor expression by these transcription factors may contribute to the tissue specificity of metabolic and immune responses.

On the other hand, not only do our results demonstrate that adhesion GPCRs with multiple binding sites for KLF14 and SREBF-1 (*ADGRB2*, *ADGRD2*, *ADGRG1*, *CELSR1*, *ADGRE2*, among others) are significantly associated with metabolic syndrome, but they are also consistent with previous studies linking these receptors to adipogenic function and glucose homeostasis [51]. However, unlike prior research, our study reveals a stronger correlation between the number of binding sites and the severity of metabolic syndrome, suggesting a potential transcriptional regulatory mechanism that could explain this association.

These findings collectively support the notion that orphan GPCRs are key players in metabolic processes and may be potential therapeutic targets for metabolic disorders.

### 3.2. Analysis of GPCRs Associated with MetS

Computational analysis identified putative binding sites for KLF14 and SREBF-1 within the promoter regions of multiple GPCRs implicated in metabolic syndrome. This suggests a potential regulatory mechanism for these transcription factors in modulating the expression of these receptors.

A majority of the GPCRs analyzed exhibited multiple binding sites for both KLF14 and SREBF-1, particularly at a *p*-value threshold of 0.01. Notably, many of these sites were in the distal promoter regions, suggesting a potential role in modulating basal expression. However, the distribution and number of binding sites varied significantly across different GPCRs, indicating a diverse regulatory landscape for KLF14 and SREBF-1.

The discovery of a high density of binding sites for the transcription factors in the distal regions of the promoters of *LEPR*, *GHSR*, *CNR1*, *HRH1*, *SIPR1*, and *P2Y6* receptors reveals a highly sophisticated transcriptional regulatory mechanism for these genes. Given that KLF14 and SREBF1 have been previously linked to metabolic syndrome, this observation suggests a direct connection between the regulation of these receptor expressions and the development of this condition. Likewise, some studies revealed and shared these findings. It was demonstrated that monogenic obesity genes are over-represented near identified loci, and several complex association signals near *LEPR* and KLF14 point towards a major contribution for common variation affecting the leptin–melanocortin system in early life [52]; *GHSR* resulted in a viable receptor for the treatment of obesity [53]; *CNR1* was linked to diabetic nephropathy [54]; a noticeable reduction in lipid accumulation was also found in extract-treated cells through the *H1R1* genes in 3T3-L1-differentiated mouse cells. In preadipocytes and adipocytes, the tested extracts showed significant alterations in various genes involved in glucose homeostasis and obesity [46].

The identified receptors play crucial roles in regulating energy homeostasis, appetite, and lipid metabolism. The identification of binding sites for KLF14 and SREBF1 in their promoters suggests that these transcription factors may play a central role in regulating these functions. Furthermore, they provide new evidence for the molecular mechanisms underlying the development of metabolic syndrome. Alterations in the expression of these receptors, due to changes in the activity of KLF14 and SREBF1, could contribute to the metabolic dysfunction characteristic of this condition.

In contrast to the other GPCRs analyzed, in no way did we find binding sites for KLF14 or SREBF-1 within the promoters of *MC4R*, *5-HT2C*, *MC3R*, *S1PR2*, *FFAR1/GPR40*, and *HCR2*. But some studies have revealed certain associations with MetS, like *MC4R* and *MC3R* in [47], *FFAR1/GPR40* in [55], and others.

This suggests that these receptors might be regulated by alternative mechanisms or transcription factors.

Collectively, these findings suggest a pivotal role of KLF14 and SREBF-1 in regulating the expression of a subset of GPCRs implicated in metabolic syndrome. The presence of multiple binding sites in the distal promoter regions indicates the potential for these transcription factors to modulate basal expression and respond to specific physiological or environmental stimuli.

A coincidence was identified in the presence of BS for KLF14 and SREBF-1 in the DR of the promoters of leptin (*LEPR*), adiponectin (*ADIPOR1*), ghrelin (*GHSR*), serotonin (*5-HT2C*), cannabinoid (*CB1/CNR1*), orexin (*OX1R/HCRTR1*), and sphingosine-1-phosphate (*S1PR1*) receptors. The convergence of regulatory elements in orphan and non-orphan metabolic receptors implies a shared functional and evolutionary history. This finding has profound implications for drug discovery as it suggests potential common targets for therapeutic intervention in the scope of metabolic diseases.

### 3.3. Statistical Analysis-Based

This study has investigated the association between the transcription factors KLF14 and SREBF-1 and the promoters of orphan GPCRs, aiming to elucidate their potential role in metabolic syndrome. Our findings provide novel insights into the molecular mechanisms underlying the regulation of these receptors and their implications for metabolic diseases.

Our results confirm a significant association between KLF14 and SREBF-1 binding sites and the promoters of orphan GPCRs. Clustering analysis revealed distinct groups of orphan GPCRs with similar binding patterns, suggesting that these receptors may be involved in shared signaling pathways relevant to metabolic processes. Notably, the density of binding sites correlated positively with gene expression levels, indicating a direct regulatory role for these transcription factors. Furthermore, the sequence similarity of KLF14 was found to modulate binding site formation, highlighting the importance of specific DNA motifs in mediating these interactions.

The negative binomial and Poisson regression analyses provided quantitative evidence for the relationship between KLF14 and SREBF-1 binding sites in the promoter region and the overall density of binding sites in the distal region. These findings indicate the following:

- KLF14: the density of KLF14 binding sites in the positive strand was significantly associated with the distal region binding sites density, even after adjusting for sequence similarity. This suggests that the presence of binding sites in the positive strand is a strong predictor of the number of binding sites in the distal region. Additionally, the sequence similarity of KLF14 significantly modulated the distal region binding site density, indicating that the structure and conservation of the KLF14 sequence are crucial for its regulatory function.
- SREBF-1: The density of SREBF-1 binding sites in the positive strand was also significantly associated with the distal region binding site density. While the sequence similarity of SREBF-1 showed a significant association, the confidence interval was wider, suggesting a potentially more complex relationship.

These findings suggest that both the quantity and quality of KLF14 and SREBF-1 binding sites in the promoter positive strand are important determinants of the distal region binding site density in the regulatory region. The combination of these factors provides a more comprehensive explanation for the regulation of orphan GPCRs expression by these transcription factors.

While our findings align with previous studies demonstrating the pivotal roles of KLF14 and SREBF-1 [56], with a strong association with High Density Lipoprotein cholesterol (HDL-C) levels, metabolic syndrome, and coronary heart disease [57,58], this research offers a novel perspective by identifying orphan GPCRs as potential targets for these transcription factors. Previous research has largely focused on the regulation of genes directly involved in lipid biosynthesis and insulin response [59,60]. However, our study expands the repertoire of genes regulated by KLF14 and SREBF-1, thereby providing a more comprehensive understanding of their regulatory networks.

A key distinction between our study and previous research lies in the statistical methods employed. While earlier studies often relied on simpler correlation analyses or Variance Analysis (ANOVA) to investigate the relationship between transcription factor binding sites and gene expression [61,62], we have employed negative binomial and Poisson regression, providing more precise estimates of model parameters and confidence intervals, leading to more robust inferences. These models can accommodate the excess variability often observed in count data, providing a more accurate representation of the underlying biological process.

By utilizing these sophisticated statistical methods, we have been able to identify a more precise and robust association between KLF14 and SREBF-1 binding sites and the density of binding sites in the distal region. Moreover, we have been able to evaluate the impact of sequence similarity on this relationship, providing deeper insights into the molecular mechanisms underlying the regulation of orphan GPCRs.

Our findings suggest that KLF14 and SREBF-1 may regulate the expression of orphan receptors through direct binding to their promoters. This binding could facilitate the recruitment of transcriptional machinery, thereby increasing the transcription rate of target genes. Additionally, KLF14 and SREBF-1 may interact with other transcription factors or genomic regulatory elements to modulate the expression of target genes.

Our analysis revealed that the relationship between KLF14 and SREBF-1 binding sites and the total number of binding sites in the DR was similar across both orphan GPCRs and GPCRs-MetS. This suggests that the regulatory mechanisms mediated by these transcription factors are likely conserved across these receptor classes.

While previous studies have hardly ever explored the role of KLF14 and SREBF-1 in the regulation of GPCR, our analysis provides novel insights into the potential similarities and differences between orphan GPCRs and GPCRs-MetS. This suggests that both receptor classes share a common regulatory landscape modulated by KLF14 and SREBF-1, which is relevant to the understanding of our results.

### 3.4. Study Implications

The Central Role of Orphan GPCRs: the study highlights the crucial role of orphan GPCRs in metabolic processes, suggesting they could be promising therapeutic targets for metabolic disorders.

Transcriptional Regulation: the identification of binding sites for KLF14 and SREBF-1 in the promoters of orphan GPCRs suggests a complex transcriptional regulatory mechanism involved in their expression.

Tissue-Specific Expression: the varying expression patterns of orphan GPCRs in different tissues and MetS models emphasize their diverse roles in metabolic regulation.

Therapeutic Potential: the findings suggest that targeting orphan GPCRs, their associated transcription factors, or the underlying regulatory mechanisms could offer novel therapeutic strategies for metabolic syndrome.

### 3.5. Study Limitations and Strengths

While this study contributes novel insights into the regulation of orphan receptors, it is important to acknowledge its limitations. The in silico nature of the analysis limits the ability to establish direct causal relationships. Further experimental validation, such as electrophoretic mobility shift assays (EMSA) and chromatin immunoprecipitation (ChIP) assays, is required to confirm the predicted protein–DNA interactions. Functional studies are necessary to analyze the specific functions of the identified orphan receptors. Additionally, the use of animal models is crucial to study the role of KLF14, SREBF-1, and orphan receptors in the development of metabolic syndrome in vivo.

However, the utilization of state-of-the-art bioinformatics tools and the analysis of a large dataset are strengths of this study.

### 3.6. Future Directions

Future research should focus on validating the findings through experimental approaches, including in vitro and in vivo studies. Analyzing the functions of orphan receptors using gain-of-function and loss-of-function techniques will provide a deeper understanding of their roles. Utilizing animal models will enable the investigation of the role of these factors in the development of metabolic diseases. Moreover, the methodology employed in this study can be adapted to investigate other transcription factors and genes, offering a broader understanding of the molecular mechanisms underlying various diseases. The goal is to develop novel therapeutic strategies based on the findings, such as designing drugs targeting orphan GPCRs or their associated transcription factors.

In general, this study has provided robust evidence for the association between the transcription factors KLF14 and SREBF-1 and the promoters of orphan receptor genes. The results suggest that these transcription factors play a crucial role in regulating the expression of these receptors and may contribute to the development of metabolic syndrome. These findings open new avenues for the development of novel therapies for the treatment of metabolic syndrome. However, further studies are required to validate these findings and elucidate the underlying molecular mechanisms.

## 4. Materials and Methods

### 4.1. Alignment Sequences Similarity-Based Process

The study aimed first to identify the remaining orphan receptors within the family of GPCRs and to carry out an alignment process with the transcription factor binding sites. To identify the orphan receptors within the GPCRs family, the International Union of Basic and Clinical Pharmacology (IUPHAR) database (IUPHAR-DB) was used, which provides in-depth coverage of the properties of G protein-coupled receptors.

The study also aimed to determine the sequence similarity between orphan receptor genes and transcription factor binding sites to identify their structures and functions, thus contributing to the overall objective. To assess the similarity between the gene sequences of orphan receptor promoters and the BS of the KLF14 and SREBF-1 TFs, an exhaustive analysis of different sequence alignment algorithms was carried out. The following category was used:

- Local alignment: the Smith–Waterman algorithm, which seeks the best local match between two sequences, regardless of their total length. This approach is useful for identifying regions of high similarity within longer sequences.

The decision-making process to use this algorithm was, above all, due to the gene sequence features (a long sequence versus a small one), a suitable characteristic for this specific algorithm.

### 4.2. Important Region Identification and Similarity

It was essential to identify important regions during the alignment process to analyze possible functions once the orphan receptor genes join the protein.

The data exploration phase involved a multi-step process to identify crucial regions within the promoter sequences of orphan receptor genes. This was achieved through the following:

Sequence retrieval and analysis

- GenBank extraction: promoters of GPCRs were extracted from the NCBI GenBank database.
- Transcription factor binding site prediction: the JASPAR database (jaspar.genereg.net) was employed to analyze these promoters for potential binding sites of the transcription factors KLF14 and SREBF-1.

Sequence alignment and comparison

- Smith–Waterman algorithm: this dynamic programming algorithm was utilized to align the promoter sequences, identifying regions of similarity and dissimilarity.
- Pearson correlation coefficient: this metric was employed to quantify the degree of similarity between sequences.
- Unweighted pair group method with arithmetic mean (UPGMA): this clustering method was used to construct a phylogenetic tree based on sequence similarities, allowing for visualization of evolutionary relationships.

Identification of important regions

- Region classification: viable binding sites were identified and categorized based on their position relative to the transcription start site, including distal regions and regions with potential enhancer or silencer activity.

By combining these techniques, the researchers aimed to uncover functional insights into the regulation of orphan receptor genes and their potential roles in cellular processes.

To evaluate heuristic methods, FASTA was employed for their computational efficiency. However, only FASTA sequences underwent dynamic programming analysis. A comprehensive bioinformatics analysis was conducted to characterize MetS and investigate orphan GPCRs. This involved:

- A thorough literature review on MetS;
- Identification of orphan GPCRs;
- Extraction of gene sequences and identification of BS using NCBI GenBank and JASPAR;
- Classification of BS into distal and regulatory regions.

The analyzed receptors were classified into three groups based on their association with TFs: Group A (KLF14), Group B (SREBF-1), and Group C (both KLF14 and SREBF-1). This classification scheme was applied to both ORs and GPCRs.

### 4.3. GPCRs-MetS Identification

At this point, it was essential to determine the GPCRs associated with MetS based on the literature review so that some conclusions could be drawn as to the expression of both groups of receptors.

To determine the GPCRs-MetS, an exhaustive review of the literature in high-impact databases was conducted, in addition to using the QIAGEN Ingenuity Pathway Analysis (IPA) QIAGEN Inc., Redwood City, CA, USA, https://digitalinsights.qiagen.com/IPA/, accessed on 12 March 2025) and the Eukaryotic Promoter Database (EPD) tools (https://epd.expasy.org/epd/, accessed on 12 March 2025). The results were classified according to the associated pathology within the MetS for subsequent analysis.

### 4.4. Clustering and PCA Statistical Analysis

At this stage, cluster identification was crucial to analyzing possible groups or receptors, considering their expression with the transcription factors.

To identify homogeneous genes, a partitional clustering analysis was performed. Before clustering, the tendency of data to form clusters was assessed using the Visual Assessment of Cluster Tendency (VAT) method. Various methods, including the elbow method, silhouette analysis, gap statistic, complete method (with the use of the Jaccard Dindex), and a method based on squared errors, were employed to determine the optimal number of clusters. Average linkage was used for hierarchical clustering, and the k-means algorithm with the Euclidean distance as the similarity metric was used for partitional clustering. Furthermore, principal component analysis (PCA) was performed to diminish data dimensionality and facilitate visualization of clusters. Clustering results were visualized using plots (ellipsoid and group distribution.

### 4.5. Regression Models for Counting Data

Finding possible differences between study variables was essential for this research. For that reason, regression models for counting data were carried out. To statistically analyze the results and confirm the hypothesis, Poisson and negative binomial regression models were fitted, respectively, to evaluate the relationship between the number of BS of the KLF14 and SREBF-1 TFs and the receptor class (orphan GPCR vs. GPCRs-MetS), with predictors such as the number of BS in the (+) strand of the gene and the similarity of the transcription factor obtained from the bioinformatic analysis. The negative binomial model was chosen because the Poisson model for KLF14 data exhibited overdispersion, so the negative binomial model was the most accurate and appropriate. The Poisson model was suitable for SREBF-1 data as it met the assumption that the mean is equal to the variance (no overdispersion).

Model fitting and selection:

- Information criteria: information criteria such as AIC and BIC were used to select the model that best fits the data.
- Vuong test: the Vuong test was employed to compare the fit of different models, especially non-nested models.

Model fit evaluation:

- Residual analysis: residuals were examined to verify if the model assumptions were met.
- Diagnostic plots: diagnostic plots were created to assess the quality of the model fit (scatter plot with smoothing lines and facets, histograms, marginal effects plots).

### 4.6. Software Packages and Libraries

To develop and fulfil the main objective, it was necessary to identify, use, and check some software packages and libraries to obtain statistical results and draw some conclusions.

Statistical software RStudio version 4.4.2 (Posit, PBC, Boston, MA, USA) was used [63] to perform the analysis using the R programming language. R packages and functions were utilized to apply appropriate statistical tests (pacman [64], MASS [65], multcomp [66], tidyverse [67], pscl [68], ggplot2 [69], rstatix [70], PupillometryR [71], GGally [72], lmtest [73], rattle [74], dendextend [75], purr [76], rgl [77], factoextra [78], ggpubr [79], clValid [80], dbscan [81], mclust [82], and functionalities were developed and executed to determine certain necessary data and statistics not yet available in the Comprehensive R Archive Network R (CRAN R) repository. All tests were performed with a significance level of 0.05 and 95% confidence interval (CI) of coefficients.

## 5. Conclusions

The study highlights the pivotal role of orphan GPCRs in metabolic processes, suggesting their potential as therapeutic targets for metabolic disorders. The identification of binding sites for KLF14 and SREBF-1 within the promoters of these receptors underscores their involvement in metabolic regulation.

Our findings unveil an intricate transcriptional regulatory network governing orphan GPCRs expression, wherein KLF14 and SREBF-1 emerge as pivotal regulators. The identification of multiple binding sites for these transcription factors within the distal promoter regions points to a sophisticated mechanism for fine-tuning basal expression levels and dynamically responding to diverse physiological cues.

By synergizing state-of-the-art bioinformatics analysis with rigorous statistical modeling, we have been able to precisely and expeditiously identify correlations between the transcription factors KLF14 and SREBF-1 and orphan receptor genes. This versatile methodology is readily adaptable to exploring the associations between other transcription factors and diverse genes and diseases.

While this study provides valuable insights, it is essential to acknowledge its limitations regarding the in silico nature of the analysis that limits the ability to establish direct causal relationships. Future studies should focus on experimental validation to confirm the interactions between KLF14, SREBF-1, and orphan GPCRs and to elucidate their functional consequences.

## Figures and Tables

**Table 1:** Binding sites and their location in important regions.

	Class A	Class B/Adhesion	Class C
Total of orphan GPCRs	85	33	8
# Binding sites	966	520	103
# Binding sites [+]	501	276	56
# Binding sites [−]	465	244	47
# Viable binding sites	501	276	56
**# important regions**			
Silencer Enhancer			
	2	20	0
	64	34	0
**Binding sites in the distal region [−750 −1000]**			
	90	49	11

**Table 2:** Distribution of binding sites (BS) among orphan receptors (Class A, B, and C) in the distal region.

	Distal Region [−750 to −1000] Upstream of the Gene Promoters Class A				
	Number of Binding Sites				
	1	2	3	4	5–10
Genes	*GPR21*	*GPR4*	*GPR45*	*GPR3*	GPR12 (8 *)
	*GPR22*	*GPR19*	*GPR78*	*GPR17*	GPR37 (5 *)
	*GPR25*	*GPR68*	*LGR4*	*GPR26*	GPR85 (5 *)
	*GPR39*	*GPR132*	*MRGPRD*	*GPR176*	
	*GPR50*	*GPR139*	*MRGPRE*	*P2RY10*	
	*GPR83*	*GPR182*	*MRGPRG*		
	*GPR84*	*MAS1*			
	*GPR135*	*P2RY8*			
	*GPR142*	*TAAR8*			
	*GPR150*	*GPR55*			
	*GPR153*				
	*GPR171*				
	*GPR183*				
	*MAS1L*				
	**Class B/Adhesion**				
	*ADGRD1*	*ADGRA1*	*ADGRB1*	*ADGRB2*	CELSR1 (5 *)
	*ADGRE1*	*CELSR3*	*ADGRB3*	*ADGRD2*	ADGRE2 (5 *)
	*ADGRF3*	*ADGRG3*	*CELSR2*	*ADGRG1*	
	*ADGRF5*	*ADGRG6*	*ADGRG5*		
	*ADGRL2*	*ADGRL1*			
	**Class C**				
	*GPR56*				GPRC5C (7 *)
	*GPR158*				
	*GPR179*				
	*GPRC5A*				

* Numbers in parentheses point out the number of BS in the orphan receptors.

**Table 3:** Number of putative binding sites for KLF14 and SREBF-1 in GPCRs promoters.

GPCR	KLF14 (*p* = 0.01)	SREBF-1 (*p* = 0.01)	DR-KLF14 (*p* = 0.01)	DR-SREBF-1 (*p* = 0.01)
*LEPR*	34	18	5	4
*ADIPOR1*	29	21	3	3
*ADIPOR2*	20	14	3	0
*GHSR*	33	9	7	4
*AGTR1*	14	19	2	5
*CNR1*	47	19	10	5
*H1R/HRH1*	36	12	7	2
*X1R/HCRTR1*	37	22	10	9
*X2R/HCRTR2*	14	11	4	4
*FFAR3/GPR41*	14	23	1	6
*S1PR1*	33	22	5	7
*P2Y6/P2RY6*	15	23	5	4

**Table 4:** GPCRs associated with metabolic syndrome and distribution of KLF14 and SREBF-1 binding sites.

Transcription Factor KLF14					
GPCRs-MetS	#BS	#BS+	#BS−	#IR+	#BS-DR
*LEPR*	50	31	19	30	1
*ADIPOR1*	6	5	1	0	1
*ADIPOR2*	1	0	1	0	0
*MC4R*	0	0	0	0	0
*GHSR*	17	4	13	0	1
*AGTR1*	2	1	1	0	0
*5-HT2C*	12	4	8	0	1
*CB1/CNR1*	9	2	7	0	1
*MC3R*	4	1	3	0	0
*H1R/HRH1*	3	1	2	0	0
*OX1R/HCRTR1*	27	12	15	6	8
*OX2R/HCRHTR2*	8	2	6	0	0
*FFAR3/GPR41*	5	2	3	0	0
*S1PR1*	32	23	9	0	11
*S1PR2*	5	3	2	0	0
*FFAR1/GPR40*	31	8	23	0	0
*HCAR2/GPR109A*	0	0	0	0	0
*P2Y6*	14	6	8	0	3
**Transcription Factor SREBF-1**					
*LEPR*	1	1	0	0	1
*ADIPOR1*	1	0	1	0	0
*ADIPOR2*	3	2	1	0	0
*MC4R*	0	0	0	0	0
*GHSR*	4	3	1	0	0
*AGTR1*	5	4	1	0	1
*5-HT2C*	4	3	1	0	0
*CB1/CNR1*	10	6	4	0	1
*MC3R*	3	1	2	0	0
*H1R/HRH1*	2	2	0	0	0
*OX1R/HCRTR1*	3	1	2	0	0
*OX2R/HCRHTR2*	7	4	3	0	0
*FFAR3/GPR41*	5	4	1	0	0
*S1PR1*	8	2	6	0	1
*S1PR2*	8	4	4	0	0
*FFAR1/GPR40*	15	9	6	0	4
*HCAR2/GPR109A*	7	6	1	0	3
*P2Y6*	6	3	3	0	2

GPCRs-MetS: GPCRs associated with metabolic syndrome; #BS: number of binding sites; #BS+: number of binding sites in the positive strand; #BS−: number of binding sites in the complementary strand; #IR+: number of binding sites in important regions of the positive strand; #BS + DR: number of binding sites in the distal region.

**Table 5:** Negative binomial regression for the number of binding sites in the distal region.

Model (Neg. Binomial)	Coefficient (Estimate)	CI (95%)	Pr (>|z|)
(Intercept)	−0.80158	(−1.1724, −0.4455)	<0.001
The number of BS in the positive strand	0.10986	(0.0776, 0.1443)	<0.001
Dispersion parameter (θ)	1.274		
Null deviance: 188.11			
Residual deviance: 136.71			
AIC: 397.39			
(Intercept)	−8.872	(−16.8515, −3.1697)	0.01247 *
klf14 similarity	10.831	(4.1175, 20.2363)	0.00951 **
Dispersion parameter (θ)	0.9107		
Null deviance: 164.37			
Residual deviance: 126.29			
AIC: 404.48			
(Intercept)	−4.10798	(−8.8290, −1.9167)	0.00946 **
The number of BS in the positive strand	0.08269	(0.0500, 0.1169)	<0.001
klf14 similarity	4.28924	(1.6102, 9.9563)	0.02437 *
Dispersion parameter (θ)	1.4771		
Null deviance: 198.51			
Residual deviance: 128.27			
AIC: 384.08			

Pr (>|z|): the probability of a more extreme z-statistic; (*): significance level less than 0.05; (**): significance level less than 0.01.

**Table 6:** Poisson regression model for the number of SREBF-1 binding sites in the distal region.

Model (Poisson)	Coefficient (Estimate)	CI (95%)	Pr (>|z|)
(Intercept)	−1.4903	(−1.8965, −1.1109)	<0.001
The number of srebf1 BS in the positive strand	0.3513	(0.2620, 0.4381)	<0.001
Null deviance: 196.92			
Residual deviance: 143.36			
AIC: 280.15			
(Intercept)	−4.245	(−8.2293, −2.1601)	0.00458 **
srebf1 similarity	4.738	(2.2484, 9.4511)	0.00771 **
Null deviance: 196.92			
Residual deviance: 164.94			
AIC: 301.74			
(Intercept)	−4.5524	(−9.5663, −2.2291)	0.0102 *
The number of srebf1 BS in the positive strand	0.2995	(0.2011, 0.3939)	<0.001
srebf1 similarity	3.9496	(1.1221, 9.8782)	0.0618 ·
Null deviance: 196.92			
Residual deviance: 132.60			
AIC: 271.39			

Pr (>|z|): the probability of a more extreme z-statistic; (*): significance level less than 0.05; (**): significance level less than 0.01; (·) denotes a *p*-value close to, but not below, the 0.05 significance threshold, indicating marginal significance.

**Table 7:** Results considering receptor groups for KLF14.

Variable	Coefficient	CI (95%)	*p*-Value
Intercept	−4.0266	(−8.7006, −1.8430)	0.0106 *
The number of klf14 BS in the positive strand	0.0744	(0.0389, 0.1119)	<0.001
Group GPCR-MetS	−0.50047	(−1.7248, 0.5975)	0.3579
Klf14 similarity	4.28399	(1.6204, 9.8874)	0.0235 *
The number of klf14 BS in the positive strand for group GPCR-MetS	−0.04647	(−0.0367, 0.1439)	0.191019

(*): significance level less than 0.05.

**Table 8:** Results considering receptor groups for SREBF-1.

Variable	Coefficient	CI (95%)	*p*-Value
Intercept	0.02943	(−0.3070, 0.3658)	0.864
The number of srebf1 BS in the positive strand	0.28852	(0.1866, 0.3903)	<0.001 ***
Group orphan GPCR-MetS	−0.40235	(−1.0964, 0.2917)	0.258
srebf1 similarity	−0.03833	(−0.5684, 0.4918)	0.888
The number of srebf1 BS in the positive strand for group orphan GPCR-MetS	0.07942	(−0.1134, 0.2722)	0.421

(***): significance level less than 0.001.

**Figure A5:**
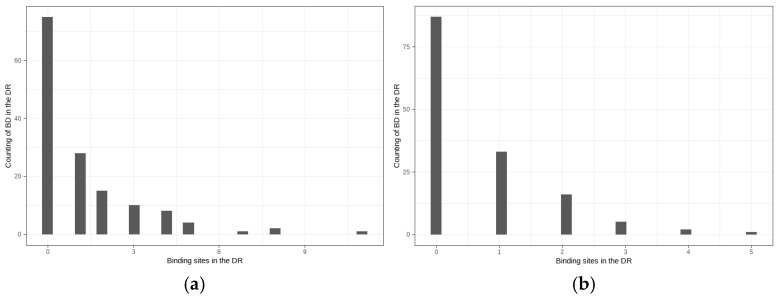
A histogram for response variable counting (**a**): BS of KLF14. (**b**): BS of SREBF-1.

**Figure A6:**
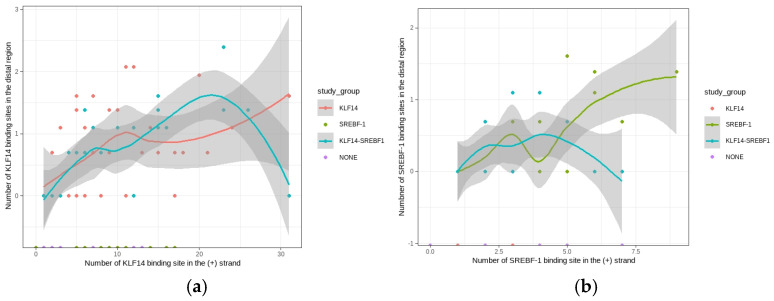
A scatter plot showing the relationship between study variables. (**a**) BS of KLF14. (**b**) BS of SREBF-1.

## Data Availability

The data presented in this study are openly available at https://jaspar.uio.no/ (accessed on 18–25 March 2024), https://www.ncbi.nlm.nih.gov/ (accessed on 7–15 May 2024), https://www.guidetopharmacology.org/GRAC/ReceptorFamiliesForward?type=GPCR (accessed on 12 July 2024). Data used to process and obtain the results shown in this study are openly available at https://drive.google.com/drive/folders/105jHtrTVUuLiYTSaSksgFKxvrqaMB1MG?usp=sharing (accessed on 12 March 2025).

## Appendix A

### Appendix A.1. Clustering Analysis for the Bioinformatics Outcomes

To illustrate the importance of this preliminary analysis, clustering was applied to two datasets: one containing genuine groups (study data) and the other randomly simulated dataset without such groups. Figure A1 confirmed the presence of genuine clusters in the study dataset, while the simulated data did not exhibit any clear grouping.

In the study dataset, a slight separation among observations can be observed, suggesting the potential formation of clusters. In contrast, the simulated dataset exhibits more compact and dispersed observations, making it more difficult to discern distinct groups. When applying clustering to both datasets (Figure A2), the formation of groups becomes slightly more evident.

Notably, the density of KLF14 BS in the DR and overall similarity were key factors in cluster formation. Group 1 exhibited a high density of distal KLF14 BS and low similarity, suggesting tighter KLF14-mediated regulation. Conversely, Group 3 showed lower distal KLF14 binding density and higher similarity.

Figure A4a shows that the ‘elbow’ point was identified around k = 4. This suggests that a clustering model with four clusters represents a suitable balance between minimizing within-cluster variance and upholding a manageable number of groups. Figure A4b presents the silhouette plot. The peak in this plot indicates that the data are optimally divided into two clusters.

Figure A4c shows the gap statistic plot. Unlike the previous methods, the gap statistics tend to increase with the number of clusters, and the error bars largely overlap.

Table A1 summarizes the proposed optimal number of clusters for the analyzed dataset, as determined by each method.

**Table A1:** The optimal number of clusters per method.

Methods	Optimal K Value
Elbow	4
Silhouette	2
Gap statistic	-
Complete	2
WBSS	6
Clusterboot	4

### Appendix A.2. Exploring Data Distribution

A histogram (Figure A5) and a scatter plot (Figure A6) were used initially to examine the possible relationship between the study variables. Figure A5 illustrates the data distribution for KLF14 and SREBF-1, showing a right-skewed distribution with a long tail. For SREBF-1, most observations had low BS counts.

Figure A6 depicts the relationship between BS in the DR and the (+) strand of KLF14 and SREBF-1. A positive correlation was observed, particularly for SREBF-1. Both KLF14 and SREBF-1 groups showed similar trends, suggesting a potential role in regulating BS in the RD. The combination of both factors (KLF14 + SREBF-1) exhibited a stronger effect, while the absence of both factors resulted in lower BS counts and a weaker trend.

### Appendix A.3. Regression Analysis for Orphan GPCRs and KLF14 and SREBF-1

#### Negative Binomial Regression Model for KLF14

Figure A7 displays the histogram of the response variable, visually confirming its count-based nature. The distribution is positively skewed, with most observations concentrated in the lower values of the BS count. Figure A8 shows a positive relationship between the variables, with a regression line indicating that as the number of BS in the (+) strand increases, so does the number of BS in the DR.

The marginal effect plot (Figure A9) shows a positive association between the number of KLF14 BS in the (+) strand and the total number of BS in the DR.

### Appendix A.4. Poisson Regression Model for SREBF-1

Figure A10 presents the marginal effects plots, illustrating how changes in one predictor modulate the expected count of BS in the DR, holding other predictors constant.
